# Jussara (*Euterpe edulis* Mart.) Supplementation during Pregnancy and Lactation Modulates the Gene and Protein Expression of Inflammation Biomarkers Induced by *trans*-Fatty Acids in the Colon of Offspring

**DOI:** 10.1155/2014/987927

**Published:** 2014-09-07

**Authors:** Carina Almeida Morais, Lila Missae Oyama, Juliana Lopez de Oliveira, Márcia Carvalho Garcia, Veridiana Vera de Rosso, Laís Sousa Mendes Amigo, Claudia Maria Oller do Nascimento, Luciana Pellegrini Pisani

**Affiliations:** ^1^Programa de Pós-Graduação Interdisciplinar em Ciências da Saúde, Universidade Federal de São Paulo, Santos, SP, Brazil; ^2^Departamento de Fisiologia da Nutrição, Escola Paulista de Medicina, Universidade Federal de São Paulo, São Paulo, SP, Brazil; ^3^Departamento de Biociências, Instituto de Saúde e Sociedade, Universidade Federal de São Paulo, Rua Silva Jardim 136, Laboratório 311, Vila Mathias, 11015-020 Santos, SP, Brazil

## Abstract

Maternal intake of *trans-*fatty acids (TFAs) in the perinatal period triggers a proinflammatory state in offspring. Anthocyanins contained in fruit are promising modulators of inflammation. This study investigated the effect of Jussara supplementation in the maternal diet on the proinflammatory state of the colon in offspring exposed to perinatal TFAs. On the first day of pregnancy rats were divided into four groups: control diet (C), control diet with 0.5% Jussara supplementation (CJ), diet enriched with hydrogenated vegetable fat, rich in TFAs (T), or T diet supplemented with 0.5% Jussara (TJ) during pregnancy and lactation. We showed that Jussara supplementation in maternal diet (CJ and TJ groups) reduced carcass lipid/protein ratios, serum lipids, glucose, IL-6, TNF-α, gene expression of IL-6R, TNF-αR (*P* < 0.05), TLR-4 (*P* < 0.01), and increase *Lactobacillus* spp. (*P* < 0.05) in the colon of offspring compared to the T group. The IL-10 (*P* = 0.035) and IL-10/TNF-α ratio (*P* < 0.01) was higher in the CJ group than in the T group. The 0.5% Jussara supplementation reverses the adverse effects of perinatal TFAs, improving lipid profiles, glucose levels, body composition, and gut microbiota and reducing low-grade inflammation in the colon of 21-day-old offspring, and could contribute to reducing chronic disease development.

## 1. Background

Variations in maternal nutrition during pregnancy and lactation may alter the physiological and morphological development of the fetus and the newborn by epigenetic modification. This process, known as metabolic programming or metabolic imprinting, can alter gene expression and permanently affect the structure and function of organs and tissues, increasing an individual's susceptibility to the development of chronic diseases [1–3].

The composition of fatty acids in the maternal diet during pregnancy and/or lactation is thus a key factor in determining whether fetal and postnatal development proceeds normally. We previously demonstrated that maternal intake of* trans-*fatty acids (TFAs), obtained industrially by partial hydrogenation of vegetable oils [4], can promote adverse effects in offspring as well as increasing the tumor necrosis factor-α (TNF-α) mRNA expression, plasminogen activator inhibitor-1 (PAI-1) mRNA expression, and TNF receptor-associated factor-6 (TRAF-6) protein in the adipose tissue of 21-day-old offspring [5, 6]. Furthermore, in adult offspring of dams fed TFAs, increased PAI-1 mRNA expression in the adipose tissue [6], increased serum endotoxin levels, NF-*κ*Bp65, TLR-4, and MyD88 protein expression, and induced hypothalamic increases in IL-6, TNF-α, and IL-1*β* [7].

Other studies have demonstrated that nutritional fatty acid exposure at perinatal stages modulates the functionality and inflammatory status of a variety of tissues and organs, including white and brown adipose tissue, skeletal muscle, and liver [5–9]. Additionally, differences in maternal dietary fat can change gut phospholipids, microbiota, intestinal permeability, and the colonic inflammatory response in offspring, particularly in animal disease models [10, 11].

Furthermore, it has been established that dietary fats modulate the gut microflora, increase colonic permeability, and trigger low-grade colon inflammation in healthy adult animals [12, 13].

The gastrointestinal tract is the first organ exposed to dietary components, and its functionality and integrity have systemic implications. In this sense, modification of microbiota, inflammation of the gut, and increases in intestinal permeability could mediate or contribute to disease development and metabolic disorders as has been proposed recently [14–16]. This process evolves with damage to the integrity of the intestinal barrier, causing an increase in bacterial translocation, and therefore an increase in the serum concentration of the external cellular membranes of gram-negative intestinal bacteria, which include lipopolysaccharide (LPS) and result in Toll-like receptor-4- (TLR-4-) mediated inflammatory responses [16–18].

LPS-induced TLR-4 provokes an inflammatory response through activation of the NF-*κ*B signaling pathway and subsequent expression of proinflammatory cytokines such as TNF-α and IL-6 [19, 20]. Likewise, elevated serum levels of free fatty acids or saturated fatty acids (SFAs) can stimulate the TLR-4 and NF-*κ*B signaling pathways and the inflammatory response [20–22].

During the perinatal period, diets rich in lipids, particularly TFAs, increase TFA-free, long-chain SFAs and decrease polyunsaturated fatty acids (PUFAs) in breast milk [23, 24]. In addition, intake of TFAs changes the lipid profile and elevates LPS serum concentration, increasing proinflammatory cytokines in offspring [5–7].

In contrast,* cis*-unsaturated fatty acids in the diet reduce the production of inflammatory cytokines and downregulate inflammation by inhibiting the NF-*κ*B signaling pathway [25, 26]. Dietary fibers, especially prebiotics, also have favorable effects on the expression of inflammatory cytokines [27, 28] by decreasing colonic pH, stimulating the gut probiotic bacterial colonization and reducing intestinal permeability and consequently the migration of LPS to circulation [18, 28–30].

Evidence has highlighted the contribution of the maternal flora to gut growth and function in the newborn. Some strains of the mother's bacterial flora are transferred through the maternal skin, fecal and vaginal contact, or breast milk [31, 32]. The transmission of maternal flora is an important variable in offspring development and health because the mother's microbiota and milk content can be affected by dietary factors [10, 32].

Foods rich in flavonoids have been identified as promising modulators of inflammation and oxidative stress [33, 34]. The fruit of the Jussara palm (*Euterpe edulis* Mart.) is a species native to the Atlantic Forest/Brazil. The fruits are rich in* cis*-unsaturated fatty acids, PUFAs, and dietary fiber and are a source of anthocyanins, flavonoids that have been shown to have high antioxidant activity, inhibit cell proliferation, and play an important role in inflammation modulation in adult animals [35–38].

Studies investigating the effect of supplementation of the maternal diet with fruits phenolics content during gestation and lactation on the inflammatory process of the offspring and the influence of early-life nutritional factors on the gut intestinal tract of healthy offspring are rare. Thus, the aim of this study was to investigate the effect of Jussara supplementation on the TFA-induced proinflammatory state in the intestinal tract of 21-day-old offspring.

## 2. Materials and Methods

### 2.1. Animals and Treatments

All experimental procedures were approved by the Experimental Research Committee of the Federal University of Sao Paulo (Protocol number 859814).

Rats were kept under controlled conditions of light (12 : 12 h light-dark cycle with lights on at 07:00) and temperature (24 ± 1°C), with* ad libitum* water and food.

Twelve-week-old female Wistar rats of first-order parity were left overnight to mate. Copulation was verified the following morning by the presence of sperm in vaginal smears. On the first day of gestation, rats were isolated in individual cages and randomly assigned to one of four groups receiving a control diet (C diet, C group), a control diet supplemented with Jussara 0.5% freeze-dried powder (CJ diet, CJ group), a diet enriched with hydrogenated vegetable fat (T diet, T group), or a T diet supplemented with 0.5% Jussara freeze-dried powder (TJ diet, TJ group).

The diets were prepared according to the recommendations of the American Institute of Nutrition (AIN-93G) [39, 40] and had similar caloric and lipid content. The source of lipids for the C and CJ diets was soybean oil; the principal source for the T and TJ diets was partially hydrogenated vegetable fat rich in TFAs. The CJ and TJ diets were prepared by adding 5 g/kg of Jussara freeze-dried powder to each diet. Jussara pulp (*Euterpe edulis* Mart.) was obtained from the agroecological Project Juçara/IPEMA—Institute of Permaculture and Ecovillages of the Atlantic (Ubatuba, SP, Brazil) and then freeze-dried to powder using a lyophilizer. Diets were then stored at −20°C. The phenolic compounds and anthocyanin contents of the Jussara pulp were previously analyzed in our laboratory [36]. The centesimal composition of the diets is presented in Table 1. The fatty acid profile of C and T diets was previously described by Pisani et al. [6].

Dams' diets were maintained during pregnancy and lactation. After birth, litter sizes were adjusted to eight pups that remained with the mother. The pups were weighed and measured (nasoanal length) at birth and on postnatal days 7, 14, and 21. After 21 days the offspring were decapitated. Trunk blood was collected and centrifuged. Serum was separated and stored at −80°C for later determination of triacylglycerol (TAG), total cholesterol, HDL-cholesterol, and glucose levels. The colon was removed and the fecal content was isolated; both were stored at −80°C.

### 2.2. Biochemical Serum Analyses

Glucose, triacylglycerol, total cholesterol, and HDL-cholesterol serum concentrations were measured with an enzymatic colorimetric method using commercial kits (Labtest Brazil).

### 2.3. Carcass Lipid and Protein Content

The carcasses were eviscerated and the remnants were weighed and stored at −20°C. The lipid content was measured as described by Stansbie et al. [41] and standardized using the method described by Oller Do Nascimento and Williamson [42]. The carcass was autoclaved at 120°C for 90 min and homogenized with water at a volume twice the carcass mass. Triplicate aliquots of approximately 3 g were digested in 3 mL of 30% KOH and 3 mL of ethanol for ≥2 h at 70°C in capped tubes. After cooling, 2 mL of 12 N H_2_SO_4_ was added and the samples were washed three times with petroleum ether to extract the lipids. The results are expressed as grams of lipid per 100 g of carcass. To measure the protein content, aliquots of the same homogenate, approximately 1 g, were heated to 37°C for 1 h in 0.6 N KOH with constant shaking. After clarification by centrifugation, protein content was measured using the Bradford assay (Bio-Rad, Hercules, CA, USA) with bovine serum albumin as a reference.

### 2.4. RNA Extraction and Real-Time Polymerase Chain Reaction (RT-PCR)

Total RNA was extracted from tissues with Tri-reagent (Sigma, St. Louis, MO, USA) and its concentration was determined from 260/280 nm absorbance ratios taken with a NanoDrop 2000/2000c (NanoDrop Technologies Inc., Wilmington, DE, USA). The TLR-4, TNF-αR, and IL-6R mRNA expression from colons were quantified by real-time polymerase chain reaction using a SYBR Green primer in StepOne Real-Time PCR Systems (Applied Biosystems, Foster City, CA, USA). Relative levels of the housekeeping gene hypoxanthine phosphoribosyl transferase (HPRT) were measured. The PCR primers used are listed in Table 2. Results were obtained using StepOne Software 2.1 (Applied Biosystems) and are expressed as a relative increase, using the method of 2^−ΔΔCt^ described by Livak and Schmittgen [43].

### 2.5. Genomic DNA Extraction from Fecal Samples and RT-PCR

Genomic DNA was extracted from colon fecal samples with the Qiagen QIAamp DNA Stool Minikit (Qiagen, Valencia, CA, USA) according to the manufacturer's recommendations. The DNA concentration per microliter was measured using the NanoDrop ND-1000 spectrophotometer (NanoDrop Technologies Inc., Wilmington, DE, USA), and the readings were acquired at wavelengths of 260, 280, and 230 nm. The purity was estimated by the 260/280 nm ratio, which must range between 1.8 and 2.0 for nucleic acids. All samples were maintained at −80°C.

### 2.6. *Lactobacillus* spp. Quantified by RT-PCR

Relative levels of* Lactobacillus *spp. DNA were quantified in real time, using a SYBR Green primer in an ABI Prism 7500 Sequence Detector (both from Applied Biosystems, Foster City, CA, USA). Relative levels of the housekeeping gene of all bacteria were measured. The PCR primers used are listed in Table 2. The results were obtained using Sequence Detector software (Applied Biosystems) and are expressed as a relative increase, using the method of 2^−ΔΔCt^, described by Livak and Schmittgen [43].

### 2.7. Colon TNF-α, IL-6, and IL-10 Protein Levels by ELISA

The colon was homogenized and centrifuged at 12,000 rpm for 40 min at 4°C; the supernatant was saved and the protein concentration determined using the BCA assay (Bio-Rad, Hercules, CA, USA) with bovine serum albumin (BSA) as a reference. Quantitative assessment of TNF-α, IL-6, and IL-10 proteins was carried out by ELISA (DuoSet ELISA, R&D Systems, Minneapolis, MN, USA) following the recommendations of the manufacturer. All samples were run as duplicates and the mean value was reported.

### 2.8. Statistical Analysis

Statistical analyses were performed using the Sigma Stat 3.5. The data were analyzed by ANOVA followed by a Bonferroni posthoc or Kruskal-Wallis test. All results are presented as the mean ± SEM and *P* ≤ 0.05 was considered statistically significant.

## 3. Results

### 3.1. Body Weight, Body Weight Gain, Length of the Animal, and Carcass Lipid and Protein Content

**Figure 1:**
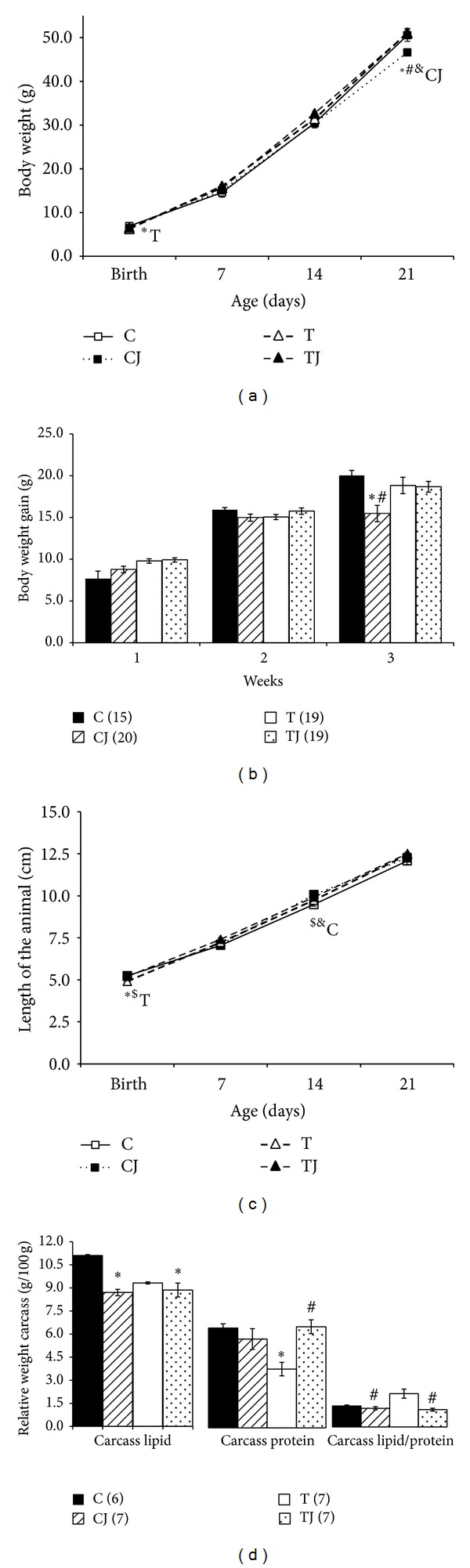
Body weight (a), body weight evolution (b), length (c), and carcass lipid, protein content, and lipid/protein ratio (d). C: offspring of dams fed control diet; CJ: offspring of dams fed control diet supplemented with 0.5% freeze-dried Jussara powder; T: offspring of dams fed diet enriched with hydrogenated vegetable fat, TFAs; TJ: offspring of dams fed diet enriched with TFAs supplemented with 0.5% freeze-dried Jussara powder. Data are means ± SEMs. The number in parentheses refers to the sample value.*P < 0.05 versus C. ^$^ *P* < 0.05 versus CJ. ^#^ *P* < 0.05 versus T. ^&^ *P* < 0.05 versus TJ.

At birth, offspring of the CJ group were longer than those of the T group (*P* < 0.05) (Figure 1(c)). The body weight (BW) and length were reduced in the pups of the T group compared to those of the C group (*P* = 0.02 and *P* < 0.05, resp.) (Figures 1(a) and 1(c)). At postnatal day 21, the length of pups did not differ between the groups and the BW of the CJ group was lower than the C (*P* = 0.042), T (*P* = 0.006), and TJ (*P* = 0.006) groups (Figure 1(a)). Furthermore, the CJ and TJ groups displayed longer body length than the C group (*P* < 0.001 and *P* = 0.017) at postnatal day 14 (Figure 1(c)). The CJ group also exhibited decreased BW gain compared with the T and C groups (*P* < 0.001) three weeks after birth (Figure 1(b)).

The relative carcass lipid levels in the CJ and TJ groups were significantly lower than in the C group (*P* < 0.05). The TJ group exhibited higher relative carcass protein levels than the T group (*P* = 0.003) and the T group contained less relative carcass protein than the C group (*P* = 0.006). The lipid to protein carcass ratio was lower in the CJ and TJ groups than in the T group (*P* < 0.05) (Figure 1(d)).

### 3.2. Biochemical Serum

The T group had increased serum concentrations of total cholesterol compared to the C group at postnatal day 21 (*P* = 0.008) while the CJ and TJ groups exhibited reduced serum levels of total cholesterol (*P* < 0.001) and glucose (*P* < 0.05) compared to the T group. Furthermore, in the TJ group the glucose concentration was lower than in the C group (*P* < 0.05). Triacylglycerol levels were also reduced in the CJ and TJ groups compared to the C and T groups (*P* < 0.05). HDL-cholesterol levels in the serum were similar in all groups (Table 3).

### 3.3. IL-6R, TNF-αR, and TLR-4 Gene Expression

**Figure 2:**
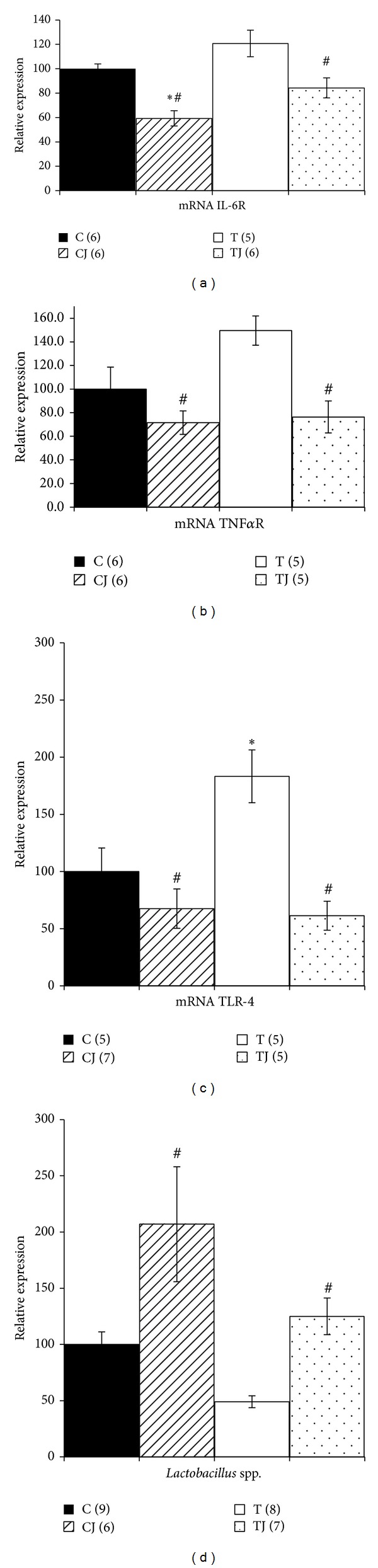
Gene expression of IL-6 receptor (IL-6R) (a), tumor necrosis factor-α receptor (TNF-αR) (b), Toll-like receptor 4 (TLR4) (c), and DNA levels of* Lactobacillus *spp. in 21-day-old offspring colon (d). C: offspring of dams fed control diet; CJ: offspring of dams fed control diet supplemented with 0.5% freeze-dried Jussara powder; T: offspring of dams fed diet enriched with hydrogenated vegetable fat, TFAs; TJ: offspring of dams fed diet enriched with TFAs supplemented with 0.5% freeze-dried Jussara powder. Data are means ± SEMs. The number in parentheses refers to the sample value. Results are expressed in arbitrary units, stipulating 100 as the control value.*P < 0.05 versus C. ^#^ *P* < 0.05 versus T.

The TLR-4 gene expression in the colon of 21-day-old offspring was higher in the T group (83.1%) than in the C group (*P* = 0.047). Levels of TNF-αR mRNA expression also increased in the T group (49.8%), but this difference was not significant. However, the CJ and TJ groups showed lower levels of TNF-αR mRNA expression (CJ group 52.1%, *P* = 0.013 versus T group; TJ group 48.9%, *P* = 0.027 versus T group) and TLR-4 mRNA expression (CJ group 63.1%, *P* = 0.002 versus T group; TJ group 66.5%, *P* = 0.002 versus T group) in offspring at postnatal day 21 (Figures 2(b) and 2(c)). In addition, the IL-6R gene expression decreased in the CJ and TJ groups compared to the T group (50.9%, *P* < 0.001; 30.2%, *P* = 0.02, resp.) and also in the CJ group compared to C group (40.7%, *P* = 0.005) (Figure 2(a)).

### 3.4. Levels of* Lactobacillus* spp. in Colon

The levels of* Lactobacillus *spp. genomic DNA in colon fecal content in the CJ and TJ groups were 4.2-fold higher and 2.6-fold higher, respectively, than in the T group (*P* < 0.05). The T group level of* Lactobacillus *spp. genomic DNA was 2.1-fold lower than the C group level; however, this difference was not significant (Figure 2(d)).

### 3.5. Cytokine Profile of the Colon

**Figure 3:**
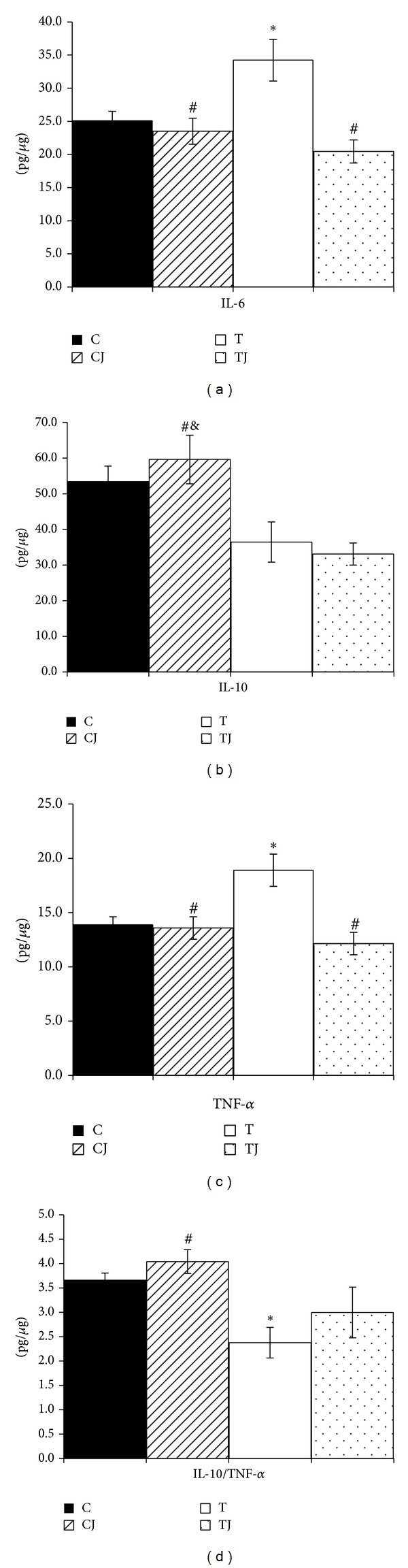
IL-6 protein expression (a), IL-10 (b), TNF-α (c), and IL-10/TNF-α ratio (d) in 21-day-old offspring colon. C: offspring of dams fed control diet; CJ: offspring of dams fed control diet supplemented with 0.5% freeze-dried Jussara powder; T: offspring of dams fed diet enriched with hydrogenated vegetable fat, TFAs; TJ: offspring of dams fed diet enriched with TFAs supplemented with 0.5% freeze-dried Jussara powder. Data are means ± SEMs of 7–11 determinations per group. *P < 0.05 versus C. ^#^ *P* < 0.05 versus T. ^&^ *P* < 0.05 versus TJ.

The protein levels of IL-6 (36.5%) and TNF-α (36.2%) were significantly higher (*P* = 0.048 and *P* = 0.013, resp.) in the T group than in the C group in offspring at postnatal day 21. However, the Jussara supplementation in the CJ and TJ groups reduced the levels of IL-6 (CJ group 31.3%, *P* = 0.011; TJ group 40.2%, *P* < 0.001) and TNF-α (CJ group 28.1% *P* = 0.013 and TJ group 35.8%, *P* < 0.001) compared to the T group (Figures 3(a) and 3(c)). Furthermore, in the CJ group, the expression of IL-10 protein was higher than in both the T group (63.4%, *P* = 0.035) and TJ group (80.2%, *P* = 0.011) (Figure 3(b)). Thus, the IL-10/TNF-α ratio in the CJ group increased compared to the T group (70.2%, *P* = 0.003) and was reduced in the T group compared to the C group (35.1%, *P* = 0.026) (Figure 3(d)).

## 4. Discussion

In this study, supplementing the maternal diet with 0.5% Jussara attenuated the adverse effects of perinatal TFAs. We showed that the maternal intake of Jussara in perinatal period modulates the inflammatory state and improves the lipid profile, glucose levels, body composition, and intestinal microbiota of 21-day-old offspring.

In our study, Jussara supplementation of the maternal diet did not affect the growth of pups at birth but in 21 day of life offspring from Jussara-supplemented dams had lower BW gain and better body composition with lower lipid content and higher carcass protein (Figure 1).

Corroborating our data, a study with açaí (*Euterpe Oleracea* Mart.) a fruit similar to Jussara, reported that supplementation with hydroalcoholic extract (200 mg/kg/day) during the pregnancy does not change BW in offspring at birth [44]. However, Rahal et al. [45] found that supplementation with 3% blueberry, which is rich in anthocyanin, to the maternal diet during pregnancy and lactation in MMTV-Wnt1-transgenic mice does not affect BW of the offspring at weaning. Furthermore, authors demonstrated that 2% Jussara supplementation in adult ApoE-deficient mice leads to no change in BW during the entire experimental period [46].

Our findings also indicate that the addition of Jussara to the maternal diet restores total cholesterol to a normal range and reduces serum TAG and glucose in 21-day-old offspring. Similar effects have been reported by De Souza et al. [47] after supplementation with 2% açaí for 6 weeks, with decreased total cholesterol observed in female Fischer rats. In fact, studies have indicated that the beneficial effect of similar fruit on the lipid profile is linked to diverse components contained in the fruit, such as* cis*-unsaturated fatty acids, polyunsaturated fatty acids (PUFAs), polyphenols, and dietary fiber. These components are associated with reduced intestinal absorption of fatty acids, greater balance in the synthesis and absorption of sterols, and increased expression of genes involved in cholesterol metabolism and excretion in the adult animals [48–50].

Moreover, there is evidence that phenolic compounds, particularly the flavonoids, induces Glut 4 in the adipose tissue and skeletal muscle and can improve glucose homeostasis and lipid metabolism via AMP-activated protein kinase activation in adult animal models [51].

Our study found that the Jussara supplementation in maternal diet led to a reduction in proinflammatory cytokines (IL-6, TNF-α) and receptors (TNFαR, IL-6R, and TLR-4 mRNA expression) induced from TFAs to normal levels, accompanied by an increase in anti-inflammatory cytokines (IL-10, IL-10/TNF-α ratio) and* Lactobacillus *spp. genomic DNA levels in the colon of offspring.

Indeed, TFAs are known for their ability to increase expression of inflammatory markers such as IL-6 and TNF-α [4, 52] and there is evidence that exposure to a high-fat diet increases inflammation in the colon [14, 15, 53] while high-polyphenol diet reduces this process [54]. Previous studies have shown that in adult offspring of mothers fed TFAs during pregnancy and lactation, high levels of LPS activate TLR-4 and mediate low-grade inflammation [7]. Additionally, as described in other studies, changes in the composition of the microbiota and the subsequent alteration of membrane permeability damage intestinal barrier integrity. This damage can cause an increase in bacterial translocation and uptake of LPS, resulting in TLR4-mediated inflammatory responses in the offspring [16, 55]. Thus, this suggests a potential mechanism by which TFAs increase the inflammatory status of the offspring colon.

In accordance with our results, we believe that the anti-inflammatory effect of Jussara also could be associated with the high nutritional value,* cis*-unsaturated fatty acids, PUFAs, bioactive compounds levels as phenolics (415 mg GAE/100 g f.m.), particularly anthocyanins (239.16 ± 7.6 mg C3R/100 g), and dietary fiber presence in the fruit of the Jussara palm [35, 36].

PUFAs can influence synthesis of the proinflammatory cytokines TNF-α and IL-6 in order to downregulate inflammatory transcription factors by actions upon intracellular signaling through the inhibition of NF-*κ*B pathway [56–58]. In this sense, the study performed by Fong et al. [59] in female rats fed a diet containing DHA, polyunsaturated long chain fatty acids (DHA group), during pregnancy and lactation, found decreased expression of TNF-α and IL-6 in 21-day-old offspring.

Likewise, polyphenols, especially anthocyanin, have been associated with the modulation of oxidative stress and inflammation in some studies from the use of similar fruits or isolation form by inhibiting NF-*κ*B activation [33, 60, 61].

Lee et al. [62] found that fruits containing different major anthocyanins showed similar anti-inflammatory effects in macrophages. Xie et al. [63] demonstrated that the diet containing 5% freeze-dried açaí (*Euterpe oleracea* Mart.) juice powder reduced TNF-α and IL-6 in adult ApoE-deficient mice model. The same authors reported that polyphenols isolated from the açaí pulp reduces these LPS-induced proinflammatory cytokines by inhibiting NF-*κ*B in macrophages [64].

The intestinal microbiota modulation has been considered as a possible mechanism by which polyphenols, particularly anthocyanins, may exert their benefic effect [65]. In recent study, high-polyphenols apple, was associated with reduction of inflammation markers and modulation in intestinal microbiota in healthy adult mice [66]. Additionally, Neyrinck et al. [67] demonstrated that the pomegranate extract, rich in phenolic compounds, modulates the gut microbiota in favor of* Bifidobacterium *spp. and downregulated IL-6 in the colon of adult mice. These authors suggest the influence of the intestinal microbiota to reduce proinflammatory cytokines by polyphenol in mice. Similarly, our findings suggest that the gut microbiota modulation have an important role in benefic effect of Jussara in offspring.

Dietary fiber has also been associated with benefic changes in intestinal microbiota, especially in the amount of bifidobacteria and lactobacilli with a consequent enhancement in colonic barrier functions [30, 68]. Increases in lactobacilli and reductions in colonic paracellular permeability have been linked to reductions in bacterial translocation and absorption of LPS, resulting in the downregulation of TLR-4-mediated inflammatory responses [16]. Recently, Arora et al. [69] demonstrated benefits in intestinal histology, reducing endotoxemia and inflammation in Female Wistar rats exposed to* Lactobacillus plantarum*. Peña and Versalovic [70] also reported an anti-inflammatory effect of* Lactobacillus rhamnosus* GG in a macrophage model, with inhibition of TNF-α production and a reduction in the TNF-α/IL-10 ratio.

Thus, it is possible that the increase in* Lactobacillus *spp. in Jussara-supplemented groups plays an important role in the downregulation of proinflammatory cytokines and the upregulation of anti-inflammatory interleukin markers in the colon of 21-day-old offspring. This effect could be associated with the fortification of the intestinal barrier integrity and intestinal mucosal permeability, which could result in reduced LPS translocation.

Therefore, we demonstrated that Jussara supplementation during pregnancy and lactation was a natural alternative to reduce of inflammation biomarkers in colon in 21-day-old offspring without altering the normality status.

## 5. Conclusion

In summary, we showed that supplementation of the maternal diet with the 0.5% Jussara during pregnancy and lactation reverses the adverse effects of perinatal TFAs. The maternal intake of Jussara in perinatal period improves lipid profiles, glucose levels, body composition, and gut microbiota and reduces low-grade inflammation in the colon of 21-day-old offspring. These effects are most likely a result of better fatty acid balance, the presence of fibers and phenolic compounds in Jussara favoring colonic bacterial population, and possibly the fortification of the intestinal barrier integrity, which could result in reduced LPS translocation. These findings support our hypothesis on the potential role of Jussara supplementation in modulating the adverse inflammatory effects of maternal TFA intake in offspring. Our results could contribute to the control of inflammation and the prevention of chronic disease development until adulthood.

## Figures and Tables

**Table 1:** Composition of the control diet (C), control diet supplemented with 0.5% freeze-dried Jussara powder (CJ), diet enriched with hydrogenated vegetable fat, TFAs (T), and diet enriched with TFAs supplemented with 0.5% freeze-dried Jussara powder (TJ) according to AIN-93.

Ingredient	C	Diet (g/100 g)		TF
		CF	T	
Casein∗	20.0	20.0	20.0	20.0
L-cystine^†^	0.3	0.3	0.3	0.3
Cornstarch^†^	62.0	62.0	62.0	62.0
Soybean oil^‡^	8.0	8.0	1.0	1.0
Hydrogenated vegetable fat^$^	—	—	7.0	7.0
Butylhydroquinone^†^	0.0014	0.0014	0.0014	0.0014
Mineral mixture^§^	3.5	3.5	3.5	3.5
Vitamin mixture^#^	1.0	1.0	1.0	1.0
Cellulose^†^	5.0	5.0	5.0	5.0
Choline bitartrate^†^	0.25	0.25	0.25	0.25
Freeze-dried Juçara powder^£^	—	0.5	—	0.5
Energy (kcal/g)	4.00	4.02	4.00	4.02

*Casein was obtained from Labsynth, São Paulo, Brazil.

^†^L-cystine, cornstarch, butylhydroquinone, cellulose and choline bitartrate were obtained from Viafarma, São Paulo, Brazil.

^‡^Oil was supplied from soybean (Lisa/Ind. Brazil).

^$^Hydrogenated vegetable fat was supplied from Unilever, São Paulo, Brazil.

^§^Mineral mix (9 mg/kg diet): calcium, 5000; phosphorus, 1561; potassium, 3600; sodium, 1019; chloride, 1571; sulfur, 300; magnesium, 507; iron, 35; copper, 6.0; manganese, 10.0; zinc, 30.0; chromium, 1.0; iodine 0.2; selenium, 0.15; fluoride, 1.00; boron, 0.50; molybdenum, 0.15; silicon, 5.0; nickel, 0.5; lithium, 0.1; vanadium, 0.1 (AIN-93G, mineral mix, Rhoster, Brazil).

^#^Vitamin mix (mg/kg diet): thiamin HCL, 6.0, riboflavin, 6.0; pyridoxine HCL, 7.0; niacin, 30.0; calcium pantothenate, 16.0; folic acid, 2.0; biotin, 0.2; vitamin B12, 25.0; vitamin A palmitate 4000 IU; vitamin E acetate, 75; vitamin D3, 1000 IU; vitamin KI, 0.75 (AIN-93G, vitamin mix, Rhoster, Brazil).

^£^Freeze-dried Juçara powder: Juçara pulp (*Euterpe edulis* Mart.) was obtained from agroecological Project Juçara/IPEMA—Institute of Permaculture and Ecovillages of the Atlantic (Ubatuba, SP, Brazil)—and by freeze-drying to powder using a lyophilizer.

**Table 2:** Nucleotide sequence of the forward and reverse primers for the RT-PCR.

Target mRNA	Forward primer	Reverse primer
HPRT	5′-CTCATGGACTGATTATGGACAGGA-3′	5′-GCAGGTCAGCAAAGAACTTATAGC-3′
TLR-4	5′-GCATCATCTTCATTGTCCTTGAGA-3′	5′-CTACCTTTTCGGAACTTAGGTCTACT-3′
TNF-*α*R	5′-GAA CAC CGT GTG TAA CTG CC-3′	5′-ATT CCT TCA CCC TCC ACC TC-3′
IL-6R	5′ AAGCAGGTCCAGCCACAATGTAG 3′	5′ CCAACTGACTTTGAGCCAACGAG 3′
All bacteria	5′-TCC TAC GGG AGG CAG CAG T-3′	5′-GAC TAC CAG GGT ATC TAA TCC TGT T-3′
*Lactobacillus *spp.	5′-AGC AGT AGG GAA TCT TCC A-3′	5′-CAC CGC TAC ACA TGG AG-3′

**Table 3:** Serum glucose, total cholesterol, HDL-cholesterol, and triacylglycerols in 21-day-old offspring.

	C (15)	CJ (20)	T (19)	TJ (19)
Glucose	110.19 ± 3.44	103.99 ± 1.73^#^	118.51 ± 2.96	98.83 ± 1.14^∗#^
Total cholesterol	120.05 ± 4.25	114.43 ± 3.57^#^	136.01 ± 2.85∗	108.79 ± 2.23^#^
HDL-cholesterol	26.56 ± 1.48	28.92 ± 1.29	25.84 ± 0.71	28.34 ± 1.03
Triacylglycerols	180.20 ± 11.37	131.11 ± 4.06^∗#^	209.19 ± 16.33	135.15 ± 3.72^∗#^

C: offspring of dams fed control diet; CJ: offspring of dams fed control diet supplemented with 0.5% freeze-dried Jussara powder; T: offspring of dams fed diet enriched with hydrogenated vegetable fat, TFAs; TJ: offspring of dams fed diet enriched with TFAs supplemented with 0.5% freeze-dried Jussara powder. Data are presented as mean ± SEM. The number in parentheses refers to the sample value.

*P < 0.05 versus C. ^$^ *P* < 0.05 versus CJ. ^#^ *P* < 0.05 versus T. ^&^ *P* < 0.05 versus TJ.
